# Diphtheria in Collins and Concord

**Published:** 1862-12

**Authors:** H. B. Horton

**Affiliations:** Eden


### DIPHTHERIA IN COLLINS AND CONCORD.

Dear Sir:—I notice in the last Journal an aacount of diphtheria prevailing in the town of Boston as reported by Dr. Davis. It has also prevailed in other sections to a very great extent, and equally severe, taking a typhoid form. It has prevailed to an alarming extent in some portions of Collins and Concord. It is not confined to the low lands or villages, but appears upon the hills and uplands, seeking its victims among the wealthy and poor alike.

H. B. Horton, M. D.

Eden, November 17th, 1862.

